# Is new dengue vaccine efficacy data a relief or cause for concern?

**DOI:** 10.1038/s41541-023-00658-2

**Published:** 2023-04-15

**Authors:** Stephen J. Thomas

**Affiliations:** SUNY Upstate Medical University, Institute for Global Health and Translational Sciences, Syracuse, NY USA

**Keywords:** Biologics, Drug development

## Abstract

Dengue is a major global public health problem requiring a safe and efficacious vaccine as the foundation of a comprehensive countermeasure strategy. Despite decades of attempts, the world has a single dengue vaccine licensed in numerous countries, but restrictions and conditions of its use have deterred uptake. Recently, clinical efficacy data has been revealed for two additional dengue vaccine candidates and the data appears encouraging. In this perspective I discuss dengue, the complexities of dengue vaccine development, early development setbacks, and how the latest data from the field may be cause for measured optimism. Finally, I provide some perspectives on evaluating dengue vaccine performance and how the pursuit of the perfect dengue vaccine may prevent advancement of vaccines which are good enough.

## Introduction

Dengue is caused by infection with any of the four dengue viruses (DENV-1–4) and represents a significant global public health burden^1^. Dengue not only causes morbidity and mortality in the infected but also consumes scarce resources for infection prevention, caring for the ill, and missed work and school^2,3^. The DENVs are transmitted in tropical and subtropical regions by infected *Aedes* mosquito species as they take a blood meal from a susceptible host. Hundreds of millions of people are infected every year and an estimated 96 million infections are clinically apparent^4,5^.

Clinically relevant dengue is characterized by fever, headache, bone and muscle pain, eye discomfort, fatigue, and the development of rash. Gastrointestinal and respiratory complaints may also be common depending on the age of the infected individual^6,7^. Severe dengue manifests with plasma leakage, intravascular volume depletion, and reduced organ perfusion (shock). Disruption of coagulation is also possible and may result in significant hemorrhage contributing to shock^8^.

Individuals are at greatest risk for severe dengue when they experience two sequential DENV infections with two different DENV types separated in time by more than 18 months^9,10^. Additional risk factors under exploration include genetic background, pre-existing medical conditions (obesity, renal and cardiovascular disease, diabetes), and female sex^11–18^. The contributions of human–vector–virus interactions and the potential evolution, and co-evolution, of human immunity, vector competence, and changes in virus genotype/lineage are also being studied^19–24^.

The exact immunopathogenic mechanisms of sequential heterotypic DENV infections are incompletely understood, but considerable evidence points to humoral and cellular adaptive immune responses occurring in response to the first infection facilitating increased DENV replication during the second infection which, in turn, drives pro-inflammatory cytokine secretion^25–31^. The exact number of annual dengue fatalities is not known but the estimates range between 5000 and 40,000 with many deaths occurring in children^32,33^.

Supportive treatment (antipyretics, judicious intravascular volume repletion) delivered by clinicians with experience treating dengue is very effective with low case fatality rates. Unfortunately, variance in clinical care exists and dengue has high morbidity and mortality in many endemic countries^34^. Currently, no anti-DENV antivirals or immune-based (monoclonal antibodies) prophylactics or therapeutics are approved for use, but promising efforts are underway^35,36^.

Reducing human arboviral infections through mosquito control strategies has had intermittent success. The widely held opinion that mosquito control is a necessary component of a comprehensive dengue control strategy requires the expanded study of available and novel approaches^37,38^. The recent development and deployment of mosquito control methods using genetic- or microbial-based alterations to mosquito populations offers the potential for improved outcomes^39,40^.

## Vaccines

Vaccination has long been recognized as the required foundation of a multi-pronged approach to reducing the global dengue burden but developing a safe and effective dengue vaccine has been very difficult. For more than 75 years, scientists and product developers have attempted to design and advance safe and efficacious dengue vaccine candidates, but the challenges have been substantial and formidable (Box 1)^41,42^. Although numerous different approaches are being explored, only live attenuated virus vaccines have achieved licensure or reached advanced clinical development^43^.

Box 1. Dengue vaccine development challenges Existence of four DENV types (1–4), each capable of causing infection, disease, and death

No validated immune correlate of protection

Animal models do not comprehensively recapitulate the human dengue infection/disease experience

Immunologic assays are unable to precisely define DENV type-specific (homotypic) immune responses

Requirement for very large efficacy trials to demonstrate benefit across diverse populations and clinical endpoints

### Dengvaxia®

Sanofi Pasteur licensed the first dengue vaccine (Dengvaxia®) in Mexico in 2015, and more than 20 countries thereafter, based on the safety and efficacy demonstrated in two phase III trials and a single season of disease surveillance. Unfortunately, the optimisim that a dengue vaccine was finally available quickly became disappointment when a safety signal was observed in vaccine recipients who were dengue non-immune at the time of vaccine administration^44,45^. In the third year of the phase III clinical trial, the youngest, non-immune vaccine recipients experienced increased rates of hospitalized and severe dengue compared to their unvaccinated peers^46^.

Many hypotheses were offered to explain this occurrence including the idea that imbalanced homotypic and heterotypic immunity across the four DENV types primed dengue naive (serostatus negative) vaccine recipients for antibody-dependant enhancement (ADE) when they encountered their first natural infection^47^. Others postulated that the absence of DENV non-structural proteins in the vaccine construct prevented the formation of protective cellular immunity and/or anti-NS1 antibodies^48^. Younger age was also proposed as an independent risk factor for clinically apparent and more severe dengue. Unfortunately, the phase III trials’ study design and limited blood sampling at baseline did not allow for a stratified analysis of vaccine safety and efficacy by baseline dengue serostatus. Instead, Sanofi tested volunteers one month after their last vaccine dose using an anti-NS1 antibody assay. The idea was that dengue serostatus negative vaccine recipients would be without NS1 antibodies because the vaccine does not contain NS1 proteins, in contrast to serostatus positive recipients who would have been naturally infected and exposed to NS1^49,50^.

The safety signal in year three became less pronounced over time but the damage was done. Sanofi had already decided to seek an indication only for older children (9 years and above) and regulators forced the company to modify the vaccine’s label stating that only individuals previously infected by a DENV should be vaccinated. There was an outcry in the Philippines as hundreds of thousands of children had been vaccinated between the time of licensure and acknowledgement of the safety signal. The country subsequently revoked the vaccine’s license.

The World Health Organization Strategic Advisory Group of Experts on Immunization (SAGE) modified its original endorsement of Dengvaxia® recommending it only be used in dengue immune individuals^51^. Although Dengvaxia was proven safe and efficacious in dengue immune recipients, especially against more severe forms of disease, and remains licensed in many countries, including the U.S., vaccination implementation and uptake has been low^52–54^. There has been little information on the outcomes, good or bad, of over 800,000 children who were vaccinated with Dengvaxia®, including hundreds of thousands who received only a single dose when the vaccination program was shut down^55^.

### The next generation of dengue vaccines

As expected, every dengue vaccine candidate following Dengvaxia® is being stringently reviewed for safety and efficacy in dengue immunes and non-immunes, across a broad age range of recipients, and for their ability to protect against the full spectrum of disease outcomes caused by infection with any DENV type. There is also a requirement for demonstrating safety and efficacy across more than one dengue season^56–58^.

Two new live attenuated dengue vaccines have now completed phase III efficacy trials and there is room for cautious optimism once again. Takeda recently received approval from Indonesia, European Commission, and Brazilian regulators for use of their two-dose vaccine (TAK-003) in people 4 years of age and older, regardless of baseline dengue immune status. Approval was based on safety, immunogenicity, and efficacy data from 19 phase I, II, and III trials with more than 28,000 participants spanning a broad age range. Dengue surveillance in the phase III trial extended for 4.5 years.

The primary study endpoint for the phase III trial was efficacy against any dengue, of any severity, caused by any DENV type in either dengue immune or non-immune recipients. Within 12 months of the second dose vaccine efficacy was 80.2%^59^. At the 18-month timepoint, vaccine efficacy against all dengue in dengue immune recipients was 76.1% and 66.2% in dengue non-immune recipients. Efficacy against hospitalized dengue was 90.4% and 85.9% against dengue hemorrhagic fever (DHF) (WHO, 1997 criteria). DENV type-specific efficacy was 69.8% for DENV-1, 95.1% for DENV-2, and 48.9% for DENV-3 with variable confidence intervals^60^. At 54 months, overall vaccine efficacy had waned to 61.2% with efficacy of 64.2% in dengue immune recipients and 53.5% in dengue non-immunes. Efficacy against hospitalized dengue was 84.1%. DENV type specific efficacy in dengue non-immunes was 78.4% for DENV-1, 100% for DENV-2, there was no efficacy for DENV-3, and not enough DENV-4 cases to calculate a value. DENV-3 efficacy in dengue immunes was 74%. Efficacy against DHF caused by any DENV type was 70.0% and against severe dengue (determined by an adjudication committee) was 70.2%. These data have not been presented in the peer-reviewed scientific literature but are accessible from the sponsor’s Summary of Product Characteristics (https://www.takeda.com/siteassets/system/newsroom/2022/qdenga/ema-combined-h-5155-en.pdf) (accessed 21 January 2023).

The dengue working group of the Centers for Disease Control and Prevention (CDC) Advisory Committee on Immunization Practices (ACIP) recently (February 23, 2023) reviewed TAK-003 performance indicating; (1) the vaccine protected seropositive recipients against all dengue and hospitalized dengue caused by infection with any serotype; (2) the vaccine protected seronegative recipients against all and hospitalized dengue due to DENV-1 and -2 infection; (3) the vaccine did not protect seronegative recipients against all dengue and hospitalized dengue due to DENV-3; and (4) the vaccine’s performance against DENV-4 infection outcomes in seronegative children could not be conclusively determined due to low event numbers. The lack of a defined immune correlate of protection made the significance of the presented immunogenicity data unclear.

More recently, the Instituto Butantan, U.S. NIH, and Merck (MSD) reported the first results from a phase III trial in Brazil with over 16,000 participants and at least two years of disease surveillance. The vaccine (Butantan-DV) was made using materials licensed from the U.S. NIH and is analogous to the NIH’s TV003 formulation tested previously^61,62^. MSD joined the collaboration when they entered into a co-development and licensing agreement in 2018. The phase III trial was initiated in 2016 and included participants ranging in age from 2 to 59 years who received a single dose of vaccine and were followed for any dengue, of any severity, caused by any DENV type. Dengue immune and non-immune participants were included in the trial.

Overall efficacy was 79.6% with dengue immunes having higher efficacy (89.2%) compared to dengue non-immunes (75.3%). Efficacy data is only available for DENV-1 (89.5%) and DENV-2 (69.6%) due to the low circulation of types DENV-3 and -4 during the trial. DENV type specific data by dengue immune status reveals higher efficacy against DENV-1 in dengue immunes (96.8%) compared to non-immunes (85.5%) and similar findings for DENV-2 (immune 83.6%, non-immune 57.9%). There were no severe cases or cases with clinical warning signs reported. The trial will continue until 2024 leaving open the possibility there will be sufficient cases caused by DENV-3 and -4 to gain a clearer view of vaccine performance against these types. These data have not been published in the peer reviewed scientific literature but are accessible from the Butantan website (https://butantan.gov.br/noticias/butantan%27s-dengue-vaccine-has-79.6-efficacy-partial-results-from-2-year-follow-up-show) (accessed 21 January 2023).

In summary, the three live attenuated dengue vaccines which have generated clinical endpoint efficacy data have all demonstrated; (1) higher efficacy in dengue immune recipients; (2) higher efficacy against more severe clinical phenotypes; (3) variance in DENV type specific efficacy, and (4) the challenge of capturing data for all desired clinical endpoints (any dengue, severe dengue, hospitalized dengue), across all DENV-1–4 types, in both dengue immune and non-immune recipients.

## Assessing dengue vaccine performance

With new dengue vaccine efficacy data becoming available, regulators, public health officials, and scientists are grappling with how to assess the risk and benefit of imperfect dengue vaccines.

### Safety

The local and systemic reactogenicity profile of a dengue vaccine must be acceptable and on par with other licensed vaccines. In addition, the rates of dengue and severe dengue cannot be greater in vaccine recipients compared to unvaccinated peers. Lack of benefit against a specific clinical outcome may be acceptable when taken in the larger context of all benefits, but associations between vaccination and developing the disease the vaccine is intended to prevent is not.

How to assess for the potential of vaccine-associated dengue is not straight forward. After two years of surveillance in the Butantan study there were no severe dengue cases nor cases with clinical warning signs. The Takeda experience, however, is more complex, and even though clinical and regulatory review committees for the European Commission and Brazil’s National Health Surveillance Agency (ANVISA) did not believe there was a safety signal in dengue non-immune recipients, this a point of contention^63–65^. Two issues may prevent achieving a consensus on the Takeda data: (1) low numbers of severe and DENV type-specific cases reducing the statistical power to make generalizable conclusions and (2) using hospitalization as a surrogate for severe disease.

One would think hospitalizing an individual is an accurate reflection of disease severity but differences in hospitalization practice across countries calls this into question. As a routine practice, trial sponsors defer to the local standards of medical care. This makes sense but presents opportunities for site-to-site variance and introduces potential confounders into data analysis. For example, some sites may admit all patients based on diagnosis alone while others only admit patients based on clinical necessity.

Markers of severe disease such as clinical signs or symptoms, or laboratory evidence of plasma leakage and/or hemorrhage would also appear to be a clear method to classify disease severity, but this approach has the potential to confound due to variance in diagnostic resources across trial sites. For example, some sites may have access to, and routinely use, ultrasound to detect fluid collections such as ascites or pleural effusions indicating the occurrence of plasma leakage. Other sites may lack these resources and must rely on less sensitive methods such as abdominal palpation or lung auscultation. Adding to the complexity of this issue is that documentation methods supporting clinical trials may not be nuanced enough to distinguish between the mere occurrence of a finding from the clinical relevance of a finding.

Even when the decision is made to utilize published classification systems of severe disease there is potential for variance. For example, vaccine developers may choose to utilize severity criteria contained within the WHO 1997 guidelines or choose the revised 2009 document^66,67^. Concern that these guidelines were designed to support clinical care and were not a good fit for use in research settings prompted the U.S. NIH to lead an effort to develop guidelines for use in interventional trials^68,69^. Sponsors may also commission external experts to develop additional criteria and guidelines like Takeda did with their dengue case adjudication committee^65^.

### Expectations based on extrapolation

Differences in vaccine construct may translate into significant qualitative differences in immune responses following vaccination. These differences should be kept in mind when predicting vaccination outcomes with newer vaccine candidates using Dengvaxia® as the reference.

**Fig. 1:**
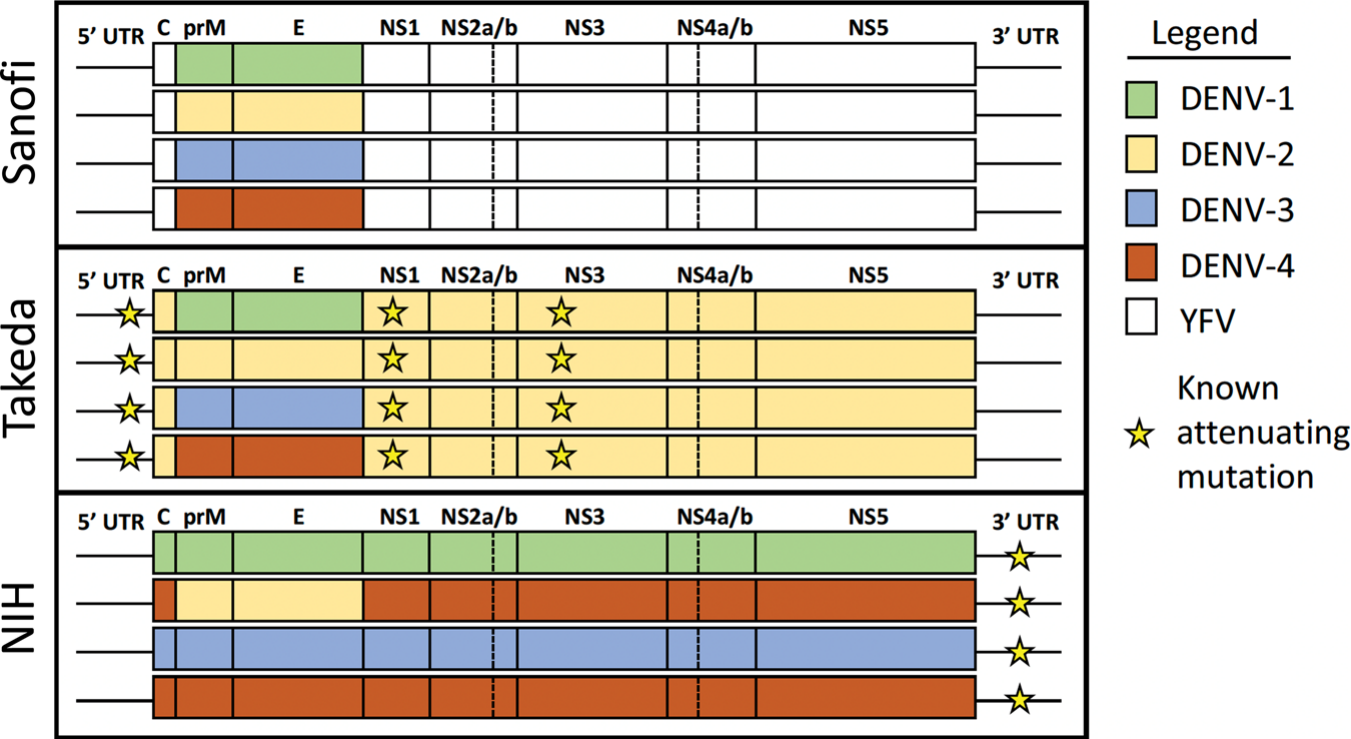
LIve attenuated dengue vaccine constructs. DENV genome components of Sanofi, Takeda, and NIH/Bhutantan/MSD dengue vaccine candidates with the location of known attenutating mutations.

Most humoral immunity epitopes are located within the domains of the DENV envelop (E) protein while cellular immunity epitopes are located on the non-structural (NS) proteins^27,70^. As noted in Fig. 1, Dengvaxia is based on a Yellow Fever (YF) 17D backbone with DENV-1-4/YF chimeras made through the introduction of DENV prM and E genes and removal of the YF prM and E genes. The vaccine contains no DENV NS proteins. The Takeda vaccine uses a DENV-2 backbone to create DENV-1/-2, -3/-2, and -4/-2 chimeras and therefore has only DENV-2 NS proteins. The NIH/Butantan/MSD vaccine has full genome DENV-1, -3, and -4 components with a DENV-2/-4 chimera based on a DENV-4 backbone. This vaccine possesses NS proteins from DENV-1, -3, and -4. These important differences in vaccine construct should motivate a pause before trying to directly and broadly extrapolate the Dengvaxia® experience to all vaccines^43^.

However, when it comes to multi-component replicating dengue vaccines, construct alone may not be sufficient to explain variance in immunogenicity and efficacy. Both the Sanofi and Takeda vaccines appear to have a dominant single vaccine virus which replicates after administration (Sanofi—DENV-4, Takeda—DENV-2) despite having all four DENV types included in the vaccine^71–73^. In contrast, MSD’s formulation of the NIH vaccine induced replication of three or more vaccine viruses in 64% of flavivirus non-immune recipients^74^. How this will translate into efficacy across the five years of follow up and DENV type-specific efficacy remains to be seen.

### Immunogenicity does not guarantee efficacy

It is unclear whether homotypic immunity to each DENV type is necessary to be protected against disease caused by infection with any DENV type. Sequential infections with two different DENV types appears to impart a mix of broadly protective homo- and heterotypic immunity, as evidenced by the very rare occurrence of clinically relevant third and fourth DENV infections^75^. Vaccine developers must pursue the development of tetravalent vaccine formulations containing antigens to each DENV type, but it has been difficult, especially with replicating vaccines, to avoid some element of immunodominance and an imbalance of homotypic immunity to the dominant DENV type and cross-reactive immunity to the others^76–82^. As a result, a major lesson learned when assessing dengue vaccine performance is that immunogenicity does not necessarily translate into clinical efficacy.

Sanofi learned this lesson following unblinding of its phase 2b efficacy trial results from Thailand^83^. The expectation was that generation of measurable neutralizing antibodies to a specific DENV type would portend a reasonable likelihood of having protection against disease if infected with the same type. But, despite having balanced geometric mean neutralizing antibody titers greater than 100 for all DENV types and high rates (>95%) of seropositivity after three vaccine doses, the trial failed to meet its primary efficacy endpoint. The immunogenicity and efficacy mismatch by DENV type would occur again during subsequent phase III testing of Dengvaxia® and Takeda’s vaccine^46,59,60,65,84–88^. Based on early efficacy data from the Butantan trial, the disconnect will likely persist based on review of the vaccine’s historic immunogenicity data and the recent disclosure of lower DENV-2 efficacy^61,89^.

### A safe and good dengue vaccine is better than no vaccine

A perfect dengue vaccine would safely deliver benefit across a myriad of scenarios. The perfect vaccine would: (1) protect across a diverse age range, (2) prevent infection (ideally) and disease caused by any DENV type and possibly numerous genotypes within each type, (3) prevent all clinically relevant phenotypes of dengue, not only severe disease, (4) protect recipients regardless of their flavivirus immunity status at the time of vaccination, (5) disrupt transmission of virus between people and mosquitoes, and (6) have durable protection until the recipient transitioned out of the risk window by acquiring a profile of homo- and heterotypic immunity like what is observed after two natural DENV infections. Shared gaps in the performance of current vaccines include a reduced ability to protect the non-immune recipient from clinically relevant, but more mild disease, caused by any DENV type.

A dengue vaccine available only to dengue immune recipients may have clinical value but lack the necessary practical attributes to support meaningful uptake. A ‘test and vaccinate’ strategy, although feasible, could be very difficult to operationalize across the multitude of dengue endemic areas^90–94^. A good vaccine not used for vaccination delivers no benefit.

Less severe forms of dengue contribute substantially to the overall public health burden^33,95–97^. Prevention of milder forms of dengue would not only reduce morbidity but also the economic and other opportunity costs of missed school or work. However, a vaccine which reliably only prevents hospitalization or more severe forms of dengue still has the potential to make a major public health impact, especially during high-transmission outbreaks (epidemics). This is particularly true in low- and middle-income countries where resources for the critically ill are scarce or in locations where experience with treating severely ill dengue patients is lacking. In addition, when hospital beds are not occupied by dengue patients, these resources can be allocated towards other public health burdens such as respiratory or gastrointestinal diseases.

A dengue vaccine that is efficacious against some, but not all DENV types can still deliver value. In many dengue endemic areas, numerous DENV types co-circulate and infect populations^98,99^. A vaccine which does not increase the recipient’s risk of dengue and can reduce the risk of disease caused by even some of the DENV types would still deliver an overall public health net benefit. This is especially true for DENV types more commonly associated with disease (DENV-1, -2) and more severe clinical outcomes (DENV-2)^11,100^.

## Conclusion

It is clear the perfect dengue vaccine is not on the immediate horizon, but the Sanofi, Takeda, and Butantan/NIH/Merck experiences do inform us that it is possible to effectively immunize some people against disease scenarios that constitute dengue’s burden. I would contend when it comes to dengue countermeasure development, safety is non-negotiable, but all other expectations must be managed and considered in the aggregate. Our challenges with effectively communicating coronavirus disease 2019 vaccine performance characteristics should be a cautionary tale in this regard. Pursuit of the perfect dengue vaccine is a laudable goal, but not at the cost of overlooking imperfect options that could safely deliver tangible, albeit smaller scale, public health benefit.
